# Performance enhancement with supplements: incongruence between rationale and practice

**DOI:** 10.1186/1550-2783-4-19

**Published:** 2007-11-12

**Authors:** Andrea Petróczi, Declan P Naughton, Jason Mazanov, Allison Holloway, Jerry Bingham

**Affiliations:** 1 School of Life Sciences, Faculty of Science, Kingston University, Penrhyn Road, Kingston upon Thames, Surrey, KT1 2EE, UK; 2 School of Business, UNSW@ADFA, Northcott Drive, Canberra ACT 2600, Australia; 3 UK Sport, 40 Bernard Street, London, WC1N 1ST, UK

## Abstract

**Background:**

Athletes are expected to consider multiple factors when making informed decision about nutritional supplement use. Besides rules, regulations and potential health hazards, the efficacy of different nutritional supplements in performance enhancement is a key issue. The aim of this paper was to find evidence for informed decision making by investigating the relationship between specific performance-related reasons for supplement use and the reported use of nutritional supplements.

**Methods:**

The 'UK Sport 2005 Drug Free Survey' data (n = 874) were re-analysed using association [χ^2^] and 'strength of association' tests [ϕ] to show the proportion of informed choices and to unveil incongruencies between self-reported supplement use and the underlying motives.

**Results:**

Participants (n = 520) reported supplement use in the pattern of: vitamin C (70.4%), creatine (36.1%), whey protein (30.6%), iron (29.8%), caffeine (23.8%), and ginseng (8.3%) for the following reasons: strength maintenance (38.1%), doctors' advice (24.2%), enhancing endurance (20.0%), ability to train longer (13.3%), and provided by the governing body (3.8%). Of thirty possible associations between the above supplements and reasons, 11 were predictable from literature precedents and only 8 were evidenced and these were not strong (ϕ < .7). The best associations were for the ability to train longer with creatine (reported by 73.9%, χ^2 ^= 49.14, p < .001; ϕ = .307, p < .001), and maintaining strength with creatine (reported by 62.6%, χ^2 ^= 97.08, p < .001; ϕ = .432, p < .001) and whey protein (reported by 56.1%, χ^2 ^= 97.82, p < .001; ϕ = .434, p < .001).

**Conclusion:**

This study provided a platform for assessing congruence between athletes' reasons for supplement use and their actual use. These results suggest that a lack of understanding exists in supplement use. There is an urgent need to provide accurate information which will help athletes make informed choices about the use of supplements.

## Background

Following deaths or near fatal incidents among high performing competitors associated with stimulant abuse [1], establishing official doping control was suggested at the 1960 Summer Olympic Games in Rome and implemented seven years later in Mexico City during a pre-Olympic event, but without having a standard testing protocol or appropriate tests available. The need for organised testing, however, was recognised and after 1973, when testing for steroids became possible, doping-testing laboratories were set up worldwide. Over the past five decades, international and national sports federations have adopted restrictions very similar to the IOC's prohibited list of substances. Under the strict regulation of performance enhancements, athletes tend to turn to nutritional supplements, which are thought to improve performance but are not prohibited by the sport governing bodies.

By general definition, nutritional supplements are products that are taken orally with the intention to supplement the diet by increasing the total dietary intake of vitamins and minerals and other non-vitamin non-mineral substances. Supplements may contain vitamins, minerals, herbs, amino acid and/or a concentrate, metabolite, constituent, extract, or a combination of any of these. In the UK, most supplements are regulated as foods and subject to the general provisions of the Food Safety Act 1990, the Food Labelling Regulations 1996 and the Trade Descriptions Act 1968. Owing to this fairly relaxed regulation, the available and heavily promoted supplements with putative performance enhancing effectiveness on the market are abundant. Manufacturers of nutritional supplements in the UK are not required to seek approval unless they are genetically modified or new supplements; or prove efficacy before marketing. Only medicinal claims on packaging are prohibited.

With contrasting advice about their use, in some cases, supplement use among elite athletes soars above 80% percent [2,3]. A recent investigation among UK junior track and field athletes [4] revealed widespread use (62%) of nutritional supplements, and that athletes are likely to use supplements in combinations (reporting an average of 2.4 supplements per athlete). Corrigan and Kazlauskas [5] analysed 2758 declaration forms from the Sydney 2000 Olympic Games doping testing procedure and found unjustified overuse of vitamins. Some athletes reported the use of up to 26 different supplements in one day [6]. Sobal & Marquart [7] report that the majority of athletes view supplement use as a requirement for good performance. The most frequently reported reasons for supplement use were compensation for poor diet, meeting the physical demands of training and competition, improving performance, keeping up with team mates and following the recommendation of a trusted person such as a parent or coach [8]. Supplements commonly ingested by athletes include antioxidants, caffeine, ephedrine, proteins, amino acids, β-Hydroxy-β-Methylbutyrate (HMB) and creatine [9].

Nutritional supplements also can have serious adverse effects if used in excessive quantity or in combinations [10,11]. Owing to diverse forms of regulation across Europe [12] and the documented widespread contamination of nutritional supplements [13], high performing athletes are generally advised by sport governing bodies to stay away from supplements as the safety of the supplements is not guaranteed. In contrast, in 2001, the American College of Sports Medicine, the American Dietetic Association, and Dieticians of Canada issued a joint position statement regarding nutrition and athletic performance [14] suggesting that physical performance and recovery are enhanced by optimal nutrition. While supplementation is normally not necessary by athletes with an adequate diet, it may be needed if energy intake is restricted or groups of food are eliminated from their diet. Some years later, a similar approach was taken in both the International Olympic Committee Consensus Statement on Sports Nutrition [15] and at the International Federation of Association Football (FIFA/F-MARC) Consensus Conference on nutrition in football [16].

The high prevalence of supplement use, coupled with the uncontrolled access to such supplements, causes concern. As nutritional supplements currently provide non-regulated alternatives to banned performance enhancing substances, their widespread use among athletes is expected. Therefore, athletes' understanding (or lack of understanding) about nutritional supplements and their efficacy should therefore be addressed and considered with the view of anti-doping prevention and interventions. Investigating nutritional supplement use for the purpose of enhancing performance is particularly important as a recent study claims the existence of a link between supplement use and doping [17]. The research among tertiary educational students (n = 2650) in six countries showed that high-risk behaviour and supplement use increased the probability of doping: those athletes who took supplements were four times more likely to use doping (2.8%) than those who used no supplements. Those who were involved in competitive sport were twice as likely to use doping as those who were not involved in competitive sport.

Therefore the aim of this study was to investigate athletes' understanding of the performance-related effects of various nutritional supplements by testing the relationship between specific performance-related reasons for supplement use and the reported use of various nutritional supplements. In regards to supplement use, we were interested in the relationship between athletes' self reported behaviour and reasoning, and not lexical knowledge.

## Methods

Data from the 'UK Sport 2005 Drug Free Survey', conducted by 20/20 Research via self-completed mail survey were used for this study [18]. Completion of the survey was voluntary and completely anonymous. Participants were aware that the results were to be published in aggregated form and gave their implied consent by returning the completed survey. As such, the project was exempt from the institutional review. Of the 2995 surveys sent, 874 were returned. The 29% response rate is in the range of the typical self-completed mail surveys.

### Data

**Figure 1:**
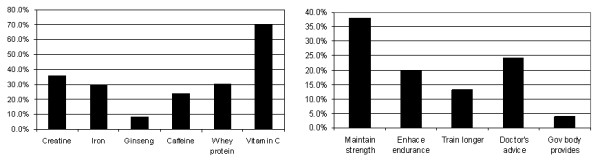
Supplements used and performance-related reasons for use by respondents who take supplements (n = 520), expressed as percentage of the sample. Respondents could select more than one reason or supplement thus the accumulated % exceeds 100%.

From the original UK Sport "Drug Free Sport" survey, answers given on two independent but related questions were analysed [18]. These questions are: 'Which supplement do you use or have you used?' and 'Why do you use supplements?', preceded by a control question regarding supplement use in general. Closed response options (a list of supplements and a list of reasons) were provided and athletes were instructed to select as many as apply and answers were coded: yes = 1, no = 0. At the end of each list, an 'other' option was offered but owing to the wide range of supplement reported, no attempt was made to categorise these answers and inclusion into the analyses. The six supplements and five parameters of performance enhancement are listed in Figure 1. Owing to inconsistencies among sport governing bodies and the lack of accurate information on which supplements are provided, if any, the '*governing body provides*' as a reason for supplement use was excluded from further analysis. Answers given to the general control question and the two probing questions provided reassurance of the validity of the data (i.e. those who claimed to use supplements also answered the two questions exploring what supplements and why were habitually used).

### Sample

Results from the 874 participants revealed that sixty percent (n = 528) of the respondents reported taking nutritional supplements, eight were deleted from further analysis because of missing data, thus the final 'supplement user' data set were reduced to n = 520. Over 30 sports are represented and respondents in the age range of 19 to 29 predominated (52.3%) followed by the age ranges of 35–39 and <18 (22% and 17%, respectively). For the supplement user sample (n = 520) relative to the entire cohort (n = 874), no significant differences were found for age group, gender or professional status. The supplement user proportion of 24–29 male professional (10% in the full sample) and 24–29 female non-professional (7% in the full sample) athletes slightly increased to 13% and 10% in the supplement user sub-sample, respectively. The proportion of the under-18 male non-professional athletes slightly decreased (from 12% to 8%) in the supplement user cohort. In summary, it can be concluded that the sample characteristics have not changed significantly after excluding the non-users. Sports represented in the sample varied greatly with European football (17.4%), badminton (14.7%), athletics (11.3%), cricket (7.2%), rugby (6.6%), swimming (6.4%) and cycling (6.2%) being dominant. The remaining 36.7% comprised 30 different sports, each contributing less than 3.5% to the overall sample.

### Analyses

Statistical analyses were performed using SPSS.14.0.1. Relationships between supplements used and motives for their use were assessed by Chi-square tests of associations and strengths of relationships [19]. Chi-square tests identify dependence between reasons for use and supplement for questions 2) and 3) above. *A ϕ *coefficient gives an estimation of the strength of the observed relationship, with *ϕ *coefficients near +1 implying responses in agreement (i.e. Yes-Yes or No-No) to both statements. Conversely, a *ϕ *coefficient near -1 shows lack of agreement in responses (i.e. Yes-No or No-Yes) to both statements. For a more detailed explanation on the method, see Petróczi and Naughton [20]. Following Rea and Parker [21], the strength of associations [*ϕ*] are categorized as strong (ϕ > .7), intermediate (.7 < ϕ > .3) and weak (ϕ < .3).

Pairs of supplements and reasons for use are given in Table 1, where literature precedents indicating expected associations [7,9,11,22-26]. These positive associations (Tables 1, 2 and 3) are expected for the following sets: creatine – *maintain strength*, *able to train longer*; iron – *enhance endurance *and *doctors' advice*; ginseng – *ability to train longer*; whey protein – *maintain strength *and *doctors' advice*; caffeine – *enhance endurance *and *able to train longer *and vitamin C – *able to train longer and doctors' advice*. In the data, the connection between a particular putative ergogenic effect and a given supplement was not explicitly made by the athlete but was calculated afterwards from answers given on the two separate and seemingly independent questions (questions 2 and 3 above). Proportions based on gender, age and profile (professional vs. non-professional) were scrutinised statistically to demonstrate that no significant differences existed for these categories between the full data set and the subset of supplement users.

**Table 1:** Summary of pairwise associations between reason for use and type of supplements used (all athletes who use supplements n = 520)

	**Maintain strength**	**Enhance endurance**	**Able to train longer**	**Doctor's advice**
**Creatine**	97.076 (< .001)	NA	49.140 (< .001)	NA
**Iron**	NA	0.230 (.632)	NA	35.004 (<.001)
**Ginseng**	NA	NA	4.062^a ^(.044)	NA
**Whey protein**	97.823 (.001)	NA	31.180 (.001)	NA
**Caffeine**	NA	38.761 (.001)	25.192 (.001)	NA
**Vitamin C**	NA	NA	1.577 (.209)	0.005 (.944)

^a ^expected association from anecdotal effect. Each cell displays the χ^2 ^coefficient and the corresponding p-values in parentheses (NA = no association expected).

**Table 2:** Summary of strength of pairwise associations between reason to use and type of supplements used (all athletes who use supplements n = 520).

	**Maintain strength**	**Enhance endurance**	**Able to train longer**	**Doctors' advice**
**Creatine**	.432 (< .001)	NA	.307 (< .001)	NA
**Iron**	NA	.021(.632)	NA	.259 (< .001)
**Ginseng**	NA	NA	.088^a ^(.044)	NA
**Whey protein**	.434 (< .001)	NA	.245 (< .001)	NA
**Caffeine**		.273 (< .001)	.220 (< .001)	NA
**Vitamin C**	NA	NA	.055 (.209)	.003 (.944)

^a ^expected association from anecdotal effect. Each cell displays the ϕ coefficient and the corresponding p-values in parentheses (NA = no association expected).

**Table 3:** Relative percentage of congruent answers by 'maintaining strength' and 'enhancing endurance' as rationales for supplement use and supplement used by all athletes who reported the use of nutritional supplements (n = 520)

		**Maintain strength**				**Enhance endurance**			
		**Yes**		**No**		**Yes**		**No**	

**Creatine**	**Yes**	124	62.6%	64	19.9%				
	**No**	74	37.4%	258	80.1%				
		198	100.0%	322	100.0%				
**Iron**	**Yes**					33	31.7%	122	29.3%
	**No**					71	68.3%	294	70.7%
						104	100.0%	416	100.0%
**Ginseng**	**Yes**								
	**No**								
**Whey protein**	**Yes**	111	56.1%	48	14.9%				
	**No**	87	43.9%	274	85.1%				
		198	100.0%	322	100.0%				
**Caffeine**	**Yes**					49	47.1%	75	18.0%
	**No**					55	52.9%	341	82.0%
						104	100.0%	416	100.0%
**Vitamin C**	**Yes**								
	**No**								

To ensure that the sample size of the UK Sport Survey data was sufficiently large for making meaningful inferences, post hoc power analysis was calculated by using the G*Power program [27]. The analysis showed that a sample size of 350 is adequate for detecting small (*w *= .15) chi-square effect [28] at a power of 80% (α = .05) and with a sample size of 500, the power of test exceeds 90% at α = .05 significance. Therefore the sample size of this study (n = 520) is adequate to detect small to medium-strong associations.

## Results

The frequencies of supplement types and reasons mentioned by supplement users are given in Figure 1. The most widely used supplement was vitamin C, used by 41.9% of all athletes in the sample. Vitamin C was taken by 70.4% of athletes who use supplements but further analysis revealed that vitamin C was not taken for performance-related reasons (Tables 1, 2, 3 and 4). The most popular performance-related supplements used were creatine and whey protein (36.1% and 30.6%, respectively) with slightly less than one third of athletes taking iron (29.8%) and/or caffeine (23.8%). A minority used ginseng (8.3%). The prevalence of creatine, whey protein, iron, caffeine and ginseng use among all athletes responded showed the same pattern (21.5%, 18.2%, 17.7%, 14.2% and 4.9%, respectively).

**Table 4:** Relative percentage of congruent answers by 'ability to train longer' and 'doctors' advice' as rationale for supplement use and supplement used by all athletes who reported the use of nutritional supplements (n = 520)

		**Able to train longer**				**Doctors' advice**			
		**Yes**		**No**		**Yes**		**No**	

**Creatine**	**Yes**	51	73.9%	137	30.4%				
	**No**	18	26.1%	314	69.6%				
		69	100.0%	451	100.0%				
**Iron**	**Yes**					64	50.8%	91	23.1%
	**No**					62	49.2%	303	76.9%
						126	100.0%	394	100.0%
**Ginseng**	**Yes**	10	14.5%	33	7.3%				
	**No**	59	85.5%	418	92.7%				
		69^a^	100.0%	451^a^	100.0%				
**Whey protein**	**Yes**	41	59.4%	118	26.2%				
	**No**	28	40.6%	333	73.8%				
		69	100.0%	451	100.0%				
**Caffeine**	**Yes**	33	47.8%	91	20.2%				
	**No**	36	52.2%	360	79.8%				
		69	100.0%	451	100.0%				
**Vitamin C**	**Yes**	53	76.8%	313	69.4%	89	70.6%	277	70.3%
	**No**	16	23.2%	138	30.6%	37	29.4%	117	29.7%
		69	100.0%	451	100.0%	126	100.0%	394	100.0%

^a ^expected association from anecdotal effect.

Analyses of the motives for supplement use reveal that of the three performance-related reasons, the desire to *maintain strength *predominates (38.1%). A notable cohort of 24.2% indicated the reasons for supplement use as *doctors' advice *and *enhancement of endurance *(20.0%) with 13.3% listing *ability to train longer *and a mere 3.8% citing *provided by governing body *as reason. Unfortunately the data did not contain information on the extent and type of supplements provided by governing bodies and if any, it was suspected to vary across sports.

The prevalence of associations are listed in Tables 1, 2, 3, and 4, and of the expected associations (11 test pairs), only eight were significant. Although expected, no significant associations were observed between iron and *enhancing endurance *or between vitamin C and *doctor's advice *or *being able to train longer*. The matrix cell for ginseng reflects the anecdotal but not-yet-proven effect of ginseng on performance.

Among reasons for supplement use, maintaining strength seems to be selected by many athletes (190/520), regardless of the sports, whereas athletes were more selective with sports specific reasons such as *endurance *(104/520) or *ability to train longer *(69/520) and there was a considerable overlap between the uses of these supplements. Of the 104 athletes who claimed *enhancing endurance *as a reason for supplement use 87 also selected *maintaining strength *from the list of reasons. The same is true for vitamin C which was taken by most athletes in the sample (366/520). For example, among those who reported the use of iron (155/520), 130 also used vitamin C; among those who used protein (159/520) 104 also reported the use of creatine.

The strength of these associations, however, varies as seen in Table 2. Of the 11 pairs with expected associations, three showed an intermediate association: *maintaining strength *– creatine and whey protein; and *ability to train longer *– creatine.

Under the assumption of a fully informed choice, it would be expected that the YY (Yes-Yes) cells to be or close to 100%. For example, those who wish to increase strength take the supplement that has this ergogenic effect (i.e. protein). However, with the exception of vitamin C, even the pairs with the strongest association coefficients showed poor results. Of those who take supplements to maintain strength, only 56.1% reported the use of whey protein. Similarly, creatine was taken by 73.9% of those who wish to be able to train longer and by only 62.6% of those who wish to maintain strength. The full matrix of the relative percentages of congruent answers by rationale for supplement use and supplement used by all athletes who reported the use of nutritional supplements are shown in Tables 3 and 4.

**Figure 2:**
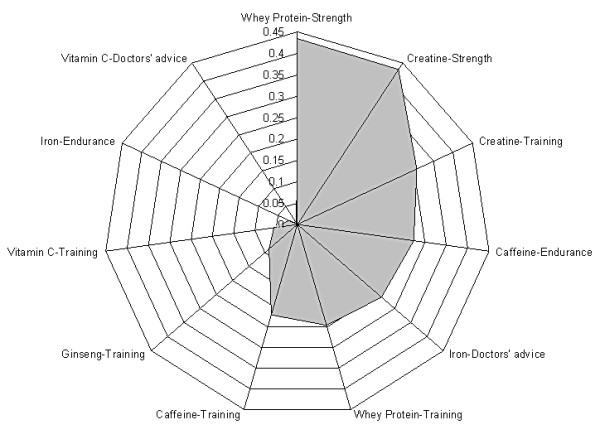
Strength of associations (expressed as phi coefficients) for the selected 9 pairs. Strength of associations are categorized as strong (ϕ > .7), intermediate (.7 < ϕ > .3) and weak (ϕ < .3).

Displaying the strength of association coefficients on a radar plot (Figure 2) it is evident that athletes are reasonably informed about supplements that can be used to help maintain strength (creatine and whey protein) but the proportion of congruent answer pairs decrease when the supplements in question are other than creatine and protein. This is not surprising as creatine has proven effects on muscle mass, high intensity exercise capacity [29] and sprint performance [30], but not on endurance or prolonged training sessions [31], and is considered as the most effective ergogenic nutritional supplement currently available to athletes [32].

## Discussion

In line with previous studies [4,5] it is apparent from this survey that athletes take a combination of substances (1674 instances for 520 users). For some pairs, reasons and supplements used show a great deal of incongruence, suggesting lack of knowledge or understanding of nutritional supplements effects.

The lack of knowledge regarding iron, vitamin C and ginseng is notable (Tables 2 and 3). While there is no clear evidence for the ergogenic properties of ginseng [24] or the much used iron [33], they are often used by athletes believing in their ergogenic properties. On the contrary, the positive effects of vitamin C on delayed muscle soreness and enhanced recovery time are well documented for [34] but these are often taken as a preventive measure in health maintenance and not for direct performance-related reasons. This is evidenced by the lack of association between performance enhancement and vitamin C (Tables 1 and 2). The relatively high percentages in Tables 3 and 4 are attributable to a 'carry-over' effect of high vitamin C use for other (i.e. health-related) reasons.

Answers regarding caffeine use were consistently negative indicating avoidance or limited use – regardless of the reason. This was despite the fact that research shows that anaerobic performance in trained athletes improves with caffeine [35], even with low doses [36]. However, low indication of caffeine use is in line with the findings of Van Thuyne and Delbeke [37] comparing caffeine concentration in urine before and after withdrawing caffeine from the WADA's prohibited substance list. It was found that the average caffeine concentrations decreased after the removal of caffeine in all sports investigated except cycling.

Responses were also inconsistent regarding medical advice and supplements used. This is a worrying sign considering that athletes seem to take supplements as per Figure 1, but many apparently do not do so because of medical advice. This result supports the concern recently raised about self-medicating, unnecessary vitamin overuse and acquired vitamin dependency [5]. Besides reasons, whether supplements are taken with or without expert advice remains unknown from these data but results definitely raise concerns that merit further investigation. From this study, doctors' advice appears to carry very little weight in terms of athletes' decisions to take supplements, which is in line with previous research that shows athletes are more likely to seek advice from coaches, peers and parents than from official sources or doctors [4,38]. This suggests that supplements are seen differently to other forms of medical intervention. According to Corrigan and Kazlauskas [5], healthy athletes do not normally need supplementation. However, athletes tend to take vitamins in excessive quantities in the (false) belief that, if a small amount helped, more could be even better [39]. Caffeine [40], vitamin C [41], and iron [42] have all been shown to have potentially adverse effects if used over a long period. Substances or methods that are not included in the current Prohibited List do not fall under the jurisdiction of the World Anti Doping Code [43], hence their use in sport in considered unregulated. The unregulated use of nutritional supplements therefore poses an increased health risk to an otherwise healthy population [44].

Only 56.1% of those who said they took supplements to increase strength indicated that they took whey protein (Table 3). One possible explanation for this phenomenon might be that respondents did not associate the product they use as being whey protein. In the UK Sport survey, athletes listed protein drinks, as well as carbohydrate and protein recovery drinks, and various product brands under 'others'. All these products contain whey protein. Soya protein was also mentioned. No evidence for association was found regarding supplements used and doctors/nutritionists' advice, with the exception of taking iron supplements.

The benefits and drawbacks of supplement use have received considerable attention in recent years, with conflicting reports frequently appearing in the literature. The contextual complexity of the supplement market and use seriously hampers the instigation of studies with robust outcomes commensurate with clinical trial approved procedures. Where reports of the observed safe levels (OSL) of individual supplements exist, they are frequently negated by the practice of intake of levels well beyond the OSL. For example, the observed safe level (OSL) for creatine (5 g/day) is frequently exceeded both in practice and in scientific studies [45]. In addition, the intake of multiple supplements is a common practice which manifests itself in both a higher number of adverse reactions and in those reactions being more severe [46]. That said, reporting side effects happens on a voluntary basis only. Regarding supplement use in athletes, further parameters under scrutiny include the interaction between medicines and supplements, the source and purity of supplements and the effects of injury or disease.

## Conclusion

This study provided a platform for assessing congruence between athletes' reasons for supplement use and their actual use. The high level of incongruence observed is alarming: athletes appear to take supplements with a poor understanding of why they are taking them. There was a clear match between only two of the most frequently-used supplements (creatine and whey protein) and the reasons given for supplement use (increase strength). There is an urgent need to provide accurate information which will help athletes make informed choices about the use of supplements. Future research should be directed to investigate supplement combinations, dose, and length of habitual supplementation as well as the source of advice athletes take regarding nutritional supplements. Expanding the list of supplements and motives would also help to gain a comprehensive view of the supplementation practices of the athlete population.

## Competing interests

The author(s) declare that they have no competing interests.

## Authors' contributions

AP conceived the study, analysed the data, drafted and revised the manuscript. DPN made contributions to the interpretation of the data, drafted and revised the manuscript. JM made contribution to the drafting and revising the manuscript. JB and AH made contribution to the acquisition and interpretation of data and revising the manuscript. All authors have read and approved the final manuscript.
